# Supplementary material for: Evidence accumulation occurs locally in the parietal cortex
Source: Nat Commun. 2022 Jul 30;13:4426. doi: 10.1038/s41467-022-32210-6 (PMC9339004; doi:10.1038/s41467-022-32210-6)
Supplement: Supplementary file 1 — Supplementary Information [file 41467_2022_32210_MOESM1_ESM.docx]

## Supplementary Information

## Supplementary Figures:


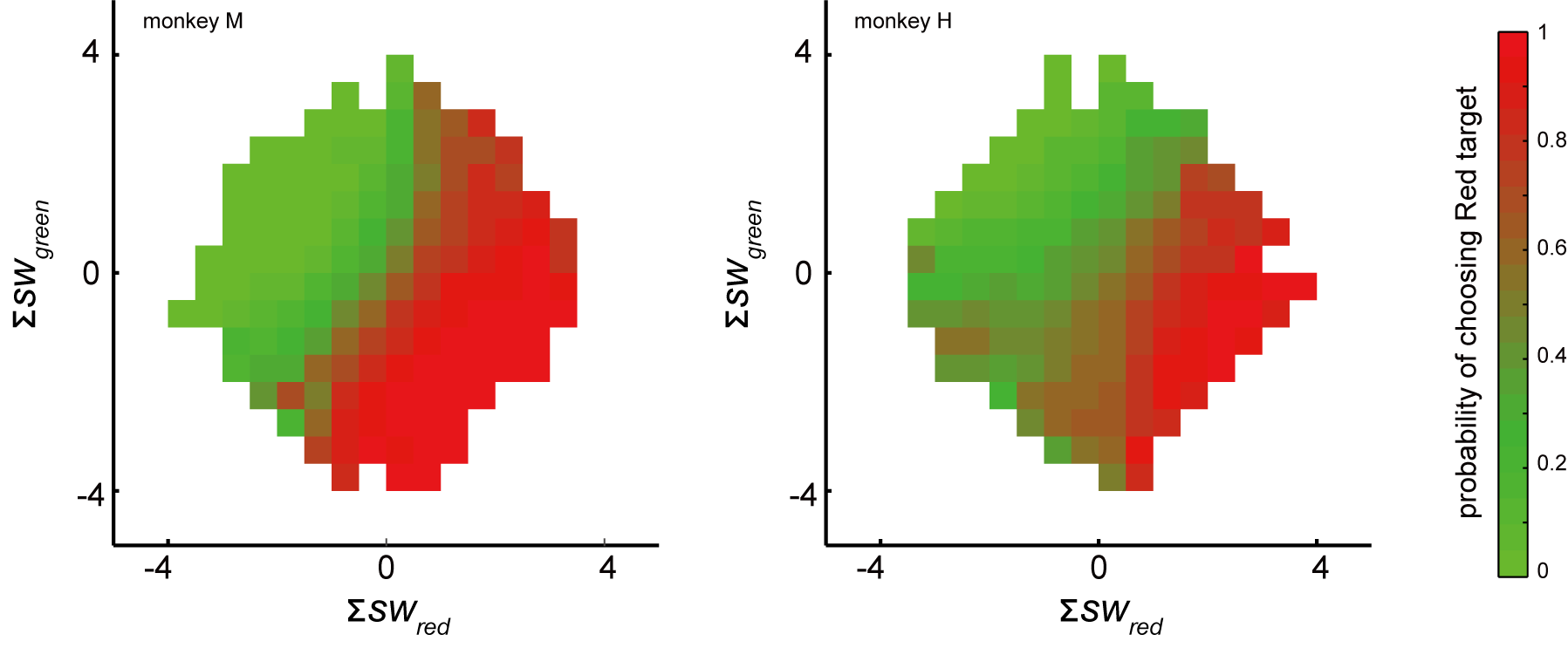


**Supplementary Figure 1**. Monkeys’ choices depended on both $\sum SW_{red}$ and $\sum SW_{green}$. Choice was plotted as a function of $\sum SW_{red}$ and $\sum SW_{green}$ for monkey M (left) and monkey H (right). Green indicates a tendency of monkey choosing the green target, red indicates the opposite tendency. Conditions with less than 10 trials are excluded. Both monkeys’ choice maps are divided diagonally, indicating the choice are best explained by the difference between $\sum SW_{red}$ and $\sum SW_{green}$.


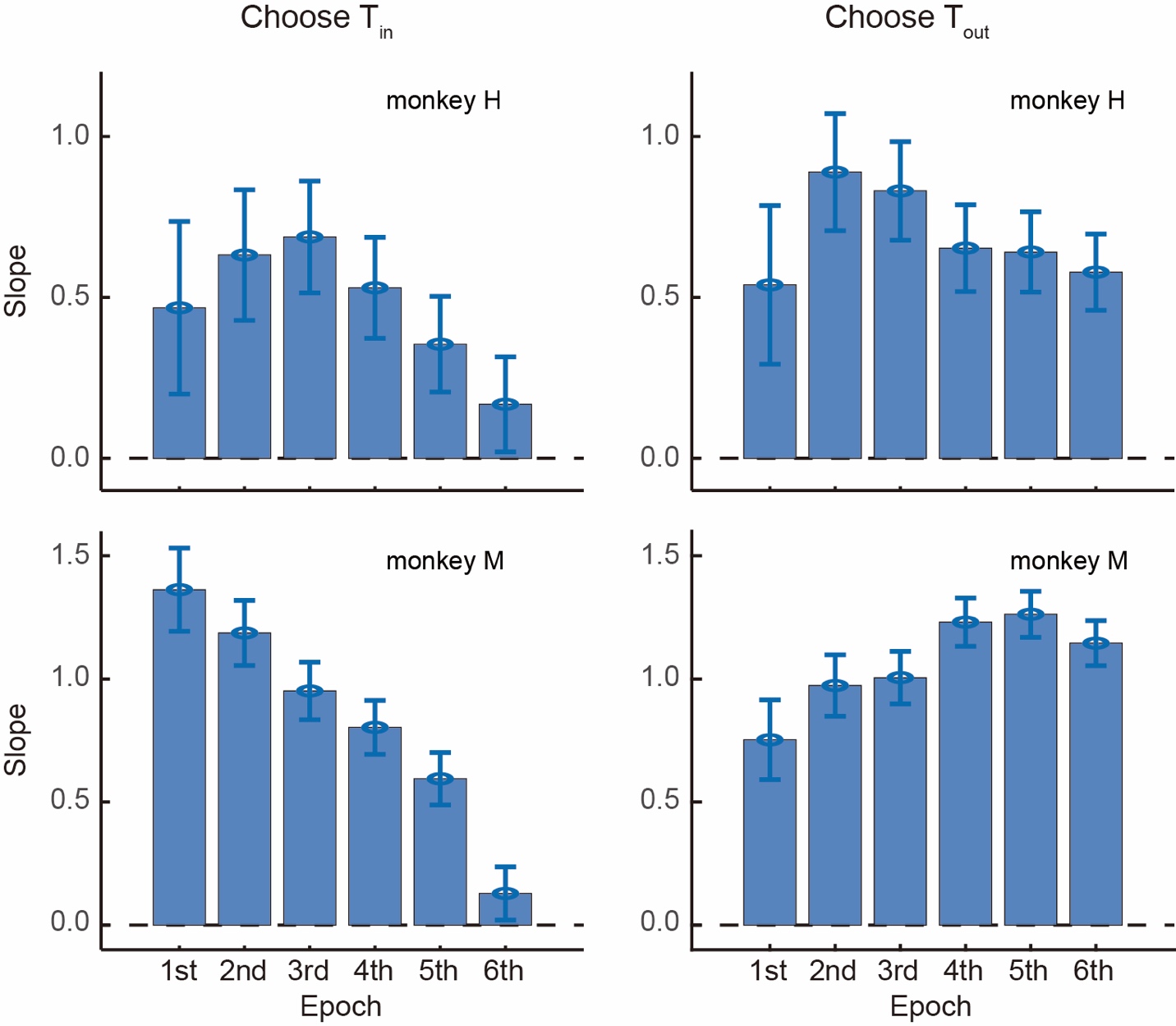


**Supplementary Figure 2**. The slopes from fitting the responses of populations of LIP neurons to the accumulated evidence (**Figure 2**, right panels), plotted for each epoch and for T_in_ (left column) and T_out_ (right column) choices separately. Data are presented as fitted slopes +/- S.E. Top row: 92 neurons from Monkey H, Bottom row: 115 neurons from Monkey M.


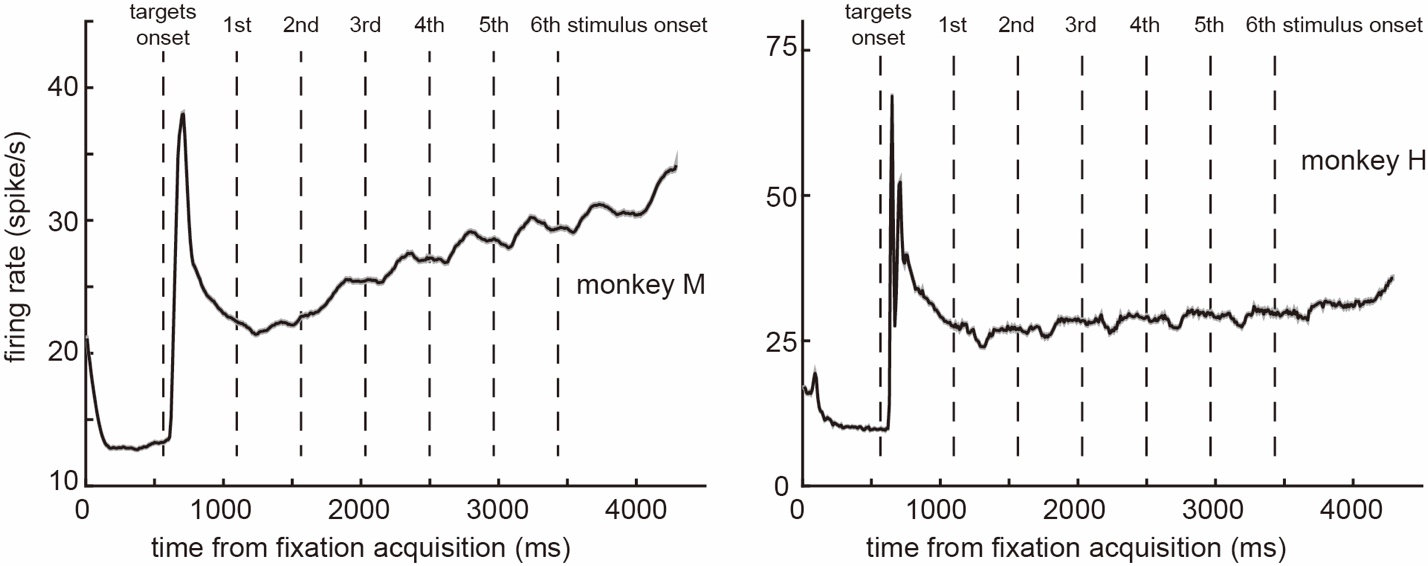


**Supplementary Figure 3**. Dips in the LIP responses. Firing rates are plotted by averaging across all trials from all neurons, combining all stimulus and choice conditions. Small dips can be seen after the onset of each shape, indicated by the vertical dash lines. Left: Monkey M, Right: Monkey H.


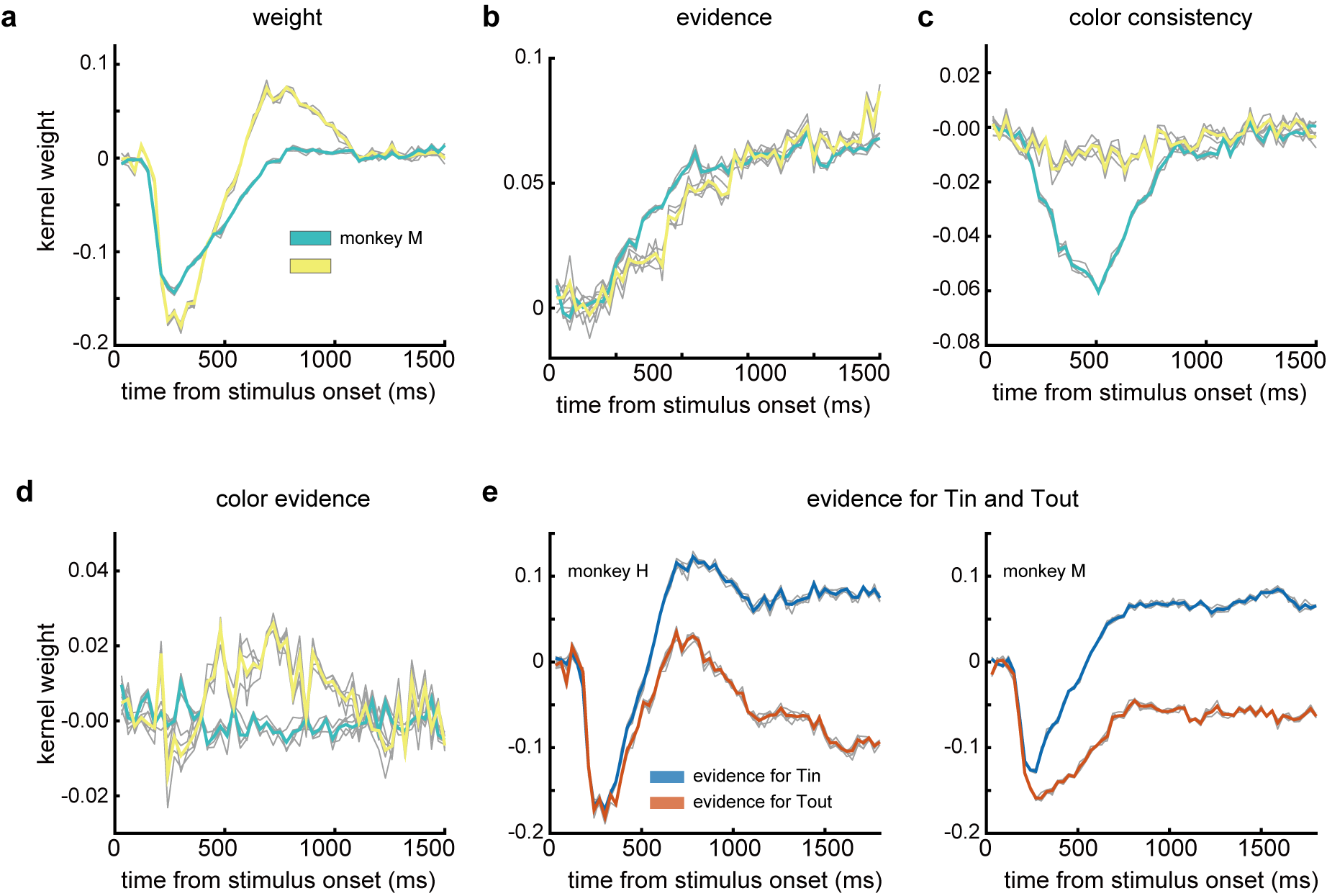


**Supplementary Figure 4**. Average kernel weights estimated from the whole data set (colored lines) and from each of the 5 cross-validation folds (grey lines). Some grey lines are overlapped and cannot be seen. **(a)** Kernel weights for the weight of shapes. **(b)** Kernel weights for the evidence. **(c)** Kernel weights for the color consistency. **(d)** Kernel weights for the color evidence. **(e)** Kernel weights for the evidence for $SW_{in}$ and $SW_{out}$.
