# Supplementary material for: Evidence accumulation occurs locally in the parietal cortex
Source: Nat Commun. 2022 Jul 30;13:4426. doi: 10.1038/s41467-022-32210-6 (PMC9339004; doi:10.1038/s41467-022-32210-6)
Supplement: Supplementary file 3 — Reporting Summary [file 41467_2022_32210_MOESM3_ESM.pdf]

nature portfolio

Corresponding author(s): Tianming Yang

Last updated by author(s): Jun 24, 2022

Reporting Summary

Nature Portfolio wishes to improve the reproducibility of the work that we publish. This form provides structure for consistency and transparency in reporting. For further information on Nature Portfolio policies, see our Editorial Policies and the Editorial Policy Checklist.

Statistics

For all statistical analyses, confirm that the following items are present in the figure legend, table legend, main text, or Methods section.

n/a	Confirmed	
☐	☒	The exact sample size (*n*) for each experimental group/condition, given as a discrete number and unit of measurement
☐	☒	A statement on whether measurements were taken from distinct samples or whether the same sample was measured repeatedly
☐	☒	The statistical test(s) used AND whether they are one- or two-sided<br>*Only common tests should be described solely by name; describe more complex techniques in the Methods section.*
☐	☒	A description of all covariates tested
☐	☒	A description of any assumptions or corrections, such as tests of normality and adjustment for multiple comparisons
☐	☒	A full description of the statistical parameters including central tendency (e.g. means) or other basic estimates (e.g. regression coefficient) AND variation (e.g. standard deviation) or associated estimates of uncertainty (e.g. confidence intervals)
☐	☒	For null hypothesis testing, the test statistic (e.g. *F*, *t*, *r*) with confidence intervals, effect sizes, degrees of freedom and *P* value noted<br>*Give P values as exact values whenever suitable.*
☒	☐	For Bayesian analysis, information on the choice of priors and Markov chain Monte Carlo settings
☒	☐	For hierarchical and complex designs, identification of the appropriate level for tests and full reporting of outcomes
☒	☐	Estimates of effect sizes (e.g. Cohen's *d*, Pearson's *r*), indicating how they were calculated

Our web collection on statistics for biologists contains articles on many of the points above.

Software and code

Policy information about availability of computer code

Data collection	MatLab 2010, MonkeyLogic Version 1, Windows 7, AlphaLab SnR Version 2.0.4.5
Data analysis	MatLab 2014, Offline Sorter Application Version 4.5.0, MatLab custom code

For manuscripts utilizing custom algorithms or software that are central to the research but not yet described in published literature, software must be made available to editors and reviewers. We strongly encourage code deposition in a community repository (e.g. GitHub). See the Nature Portfolio guidelines for submitting code & software for further information.

Data

Policy information about availability of data

All manuscripts must include a data availability statement. This statement should provide the following information, where applicable:

- Accession codes, unique identifiers, or web links for publicly available datasets
- A description of any restrictions on data availability
- For clinical datasets or third party data, please ensure that the statement adheres to our policy

The data collected in this study have been deposited in the Zenodo database and are accessible at http://doi.org/10.5281/zenodo.6555461. (the link will only be valid when a publication date is provided)

Field-specific reporting

Please select the one below that is the best fit for your research. If you are not sure, read the appropriate sections before making your selection.

☒ Life sciences ☐ Behavioural & social sciences ☐ Ecological, evolutionary & environmental sciences

For a reference copy of the document with all sections, see nature.com/documents/nr-reporting-summary-flat.pdf

Life sciences study design

All studies must disclose on these points even when the disclosure is negative.

Sample size	A total of 207 LIP neurons were collected and analyzed in two monkeys. Most monkey electrophysiology studies follow the convention of using two animals.
Data exclusions	Neurons without persistent activity and spatial selectivity in a memory saccade task were excluded. Session with fewer than 300 trials were excluded.
Replication	Following the convention of the field, we replicated the experiments and analyses in two animals. The main conclusions were replicated in both animals, and the differences are described and explained in the manuscript.
Randomization	The animals were chosen based on availability. The conditions were pseudo-randomized in each trial. The neurons were chosen based on availability.
Blinding	Not possible for data collection. Only one experimenter collected data for most of the time and analysed all data

Reporting for specific materials, systems and methods

We require information from authors about some types of materials, experimental systems and methods used in many studies. Here, indicate whether each material, system or method listed is relevant to your study. If you are not sure if a list item applies to your research, read the appropriate section before selecting a response.

Materials & experimental systems

n/a	Involved in the study	
☒	☐	Antibodies
☒	☐	Eukaryotic cell lines
☒	☐	Palaeontology and archaeology
☐	☒	Animals and other organisms
☒	☐	Human research participants
☒	☐	Clinical data
☒	☐	Dual use research of concern

Methods

n/a	Involved in the study	
☒	☐	ChIP-seq
☒	☐	Flow cytometry
☒	☐	MRI-based neuroimaging

Animals and other organisms

Policy information about studies involving animals; ARRIVE guidelines recommended for reporting animal research

Laboratory animals	Two male rhesus macaques (10-11 kg, 1-8 YO)
Wild animals	No wild animals were used in this study.
Field-collected samples	No field collected samples were used in this study.
Ethics oversight	All experimental methods were approved by the Animal Care Committee of Shanghai Institutes for Biological Sciences, Chinese Academy of Sciences.

Note that full information on the approval of the study protocol must also be provided in the manuscript.
